# Relationship between structural changes, hydrogen content and annealing in stacks of ultrathin Si/Ge amorphous layers

**DOI:** 10.1186/1556-276X-6-189

**Published:** 2011-03-01

**Authors:** Cesare Frigeri, Miklós Serényi, Nguyen Quoc Khánh, Attila Csik, Ferenc Riesz, Zoltán Erdélyi, Lucia Nasi, Dezső László Beke, Hans-Gerd Boyen

**Affiliations:** 1 CNR-IMEM Institute, Parco Area delle Scienze 37/A, 43100 Parma, Italy; 2 Research Institute for Technical Physics and Materials Science, Hungarian Academy of Sciences, P.O. Box 49, H-1525 Budapest, Hungary; 3 Institute of Nuclear Research of the Hungarian Academy of Sciences, P.O. Box 51, H-4001 Debrecen, Hungary; 4 Department of Solid State Physics, University of Debrecen, P.O. Box 2, H-4010 Debrecen, Hungary; 5 Institute for Materials Research (IMO), Hasselt University, Diepenbeek, Belgium

## Abstract

Hydrogenated multilayers (MLs) of a-Si/a-Ge have been analysed to establish the reasons of H release during annealing that has been seen to bring about structural modifications even up to well-detectable surface degradation. Analyses carried out on single layers of a-Si and a-Ge show that H is released from its bond to the host lattice atom and that it escapes from the layer much more efficiently in a-Ge than in a-Si because of the smaller binding energy of the H-Ge bond and probably of a greater weakness of the Ge lattice. This should support the previous hypothesis that the structural degradation of a-Si/a-Ge MLs primary starts with the formation of H bubbles in the Ge layers.

## Introduction

Hydrogenated a-Si and a-Ge layers are key materials for employment in (nano) structures used, e.g., in the technology of multi-junction solar cells as a-Ge acts as the low-band gap absorber while a-Si acts as the high-band gap one, thus allowing a better exploitation of the solar spectrum and the achievement of higher efficiencies [1]. However, the a-SiGe alloy is now the material of choice as the low-band gap absorber [2-4]. It allows a higher degree of freedom as regards the choice of the band gap, as the latter one can be tailored over some range by changing the Si/Ge ratio [2,4]. The a-SiGe alloy can be realized from a sequence of thin a-Si and a-Ge layers by intermixing them [1,5,6], which is obtained by heat treatments. The latter treatments are often also used for activating dopants.

**Figure 1:**
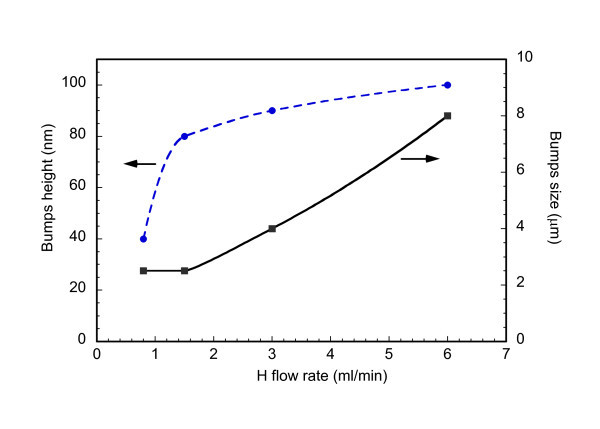
**Bumps height (dash blue line) and size (solid black line) as a function of the H flow rate in a-Si/a-Ge ML samples annealed at 350°C for 10 h**.

Previous studies have shown that the hydrogen content and annealing conditions can dramatically influence the structural stability of the a-Si/a-Ge multilayers (MLs) produced by sputtering and then annealed to produce intermixing [7-9]. It was reported that surface bumps formed, size and height of which increased with increasing H content and/or annealing temperature and time [7-9] (see Figure 1). Craters also formed subsequent to the explosion of the bumps. The bumps were ascribed to the formation of bubbles of hydrogen in the MLs [7,8]. The formation of H bubbles was also suggested by Acco et al. [10] in single layers of a-Si. It was hypothesized that H could be first released from the Ge layers because of the lower binding energy of the Ge-H bond with respect to the Si-H one [7,8]. To check this hypothesis, the MLs were additionally investigated by IR absorbance and an analysis of the structural behaviour of single films of a-Si and a-Ge, submitted to the same annealing as for the MLs previously studied, was performed. The results are reported in this article.

## Experiment

The investigated samples were MLs of alternating layers of a-Si and a-Ge and single layers of a-Si and of a-Ge. The latter ones had a thickness of 40 nm. In the former structure, the 2 × 50 alternating layers were 3 nm thick each. Both types were sputtered from high-purity crystalline silicon and germanium targets in a conventional high-vacuum sputtering apparatus (Leybold Z400) pumped to a base pressure better than 5 × 10^-5 ^Pa. The target was coupled to a RF generator (13.56 MHz) by a network for impedance matching between the generator and its load. As substrate, polished (100) Si wafers mounted on a water-cooled stage 50 mm away from the target were used. The substrate temperature was ≤60°C. It was estimated from measurements of the shift of the emission spectra of InP or GaAs during the deposition of AR (anti reflection) coating for laserdiode, carried out under identical conditions as those used here, by applying the rule 4 nm = 10°C. The temperature increase was always ≤40°C. Sputtering was done with a mixture of high-purity argon and hydrogen gases. No pressure fluctuation was observed. Plasma pressure of 2 Pa and a 1500 V dc wall potential were applied to sputter the targets, yielding a sputtering rate of 6.3 and 13.5 nm/min for a-Si and a-Ge, respectively. Hydrogenation was carried out by letting hydrogen flow into the deposition chamber at flow rates of 0.4, 0.8 and 1.5 ml/min. These values correspond to the measured 0.38, 0.78 and 1.46% partial of total pressure, respectively (all gauge readings were corrected by gas-sensitivity factors). The samples were annealed in high-purity (99.999%) argon at 350 or 400°C for 1, 4 and 10 h. The choice of such temperatures as the optimal ones for the purpose of this experiment was suggested by the findings of previous studies [7-9]. In fact, it was observed that annealing at 450°C causes such a great degradation of the surface that nearly 53% of it was covered by bumps and craters as large as 9 μm, with the craters as deep as the whole ML [7,8]. On the other hand, for annealing temperature of 250°C the formation of the craters rarely occurs only for very high H flow rate (6 ml/min) [7,8], thus making it difficult to evaluate whether the decrease of H content in the samples can be associated with craters for the lower H flow rates considered here. No crater was ever detected for annealing temperatures lower than 250°C. Non-hydrogenated samples were also sputtered to use as reference samples; they were annealed under the same conditions as the hydrogenated ones.

The samples were analysed by elastic recoil detection analysis (ERDA), atomic force microscopy (AFM), infrared (IR) absorption and Makyoh topography (MT). For ERDA, the 1.6 MeV ^4^He^+ ^beam available at the 5 MeV Van de Graaff accelerator (Research Institute for Nuclear and Particles Physics, Budapest, Hungary) had been applied to measure the hydrogen in the samples. The hydrogen recoiled from the sample by He ions was collected by surface-biased Si detector placed at a detecting angle of 10° with regard to the beam direction, while the sample was tilted to 85° from the normal. Mylar foil with thickness of 6 μm was placed in front of the ERDA detector to stop the forward-scattered He ions. Therefore, the ERDA spectra of the H are almost background-free. Low ion current (ca. 6 nA) has been used to avoid beam heating, i.e. the escape of H from the sample at a high temperature. Evaluation of ERDA spectra was done by the RBX program developed by Kótai [11]. The in-depth spatial resolution of ERDA is approximately 20 nm [12]. Since ERDA is applied here only to the single layers and the Si substrates do not contain H, such error on the depth where the ERDA signal comes from does not impair the results regarding the presence and concentration of H. The region between channels 120 and 100 (see next section) corresponds to a depth of 40 nm from the surface. The relative error on concentration is a few per cent. Therefore, the method is suitable with regard to detecting the tendency of the H change in the samples as presented in the next section. However, the absolute error is worse because of the lack of a calibration sample having a well-known H content. A carbon layer containing H was thus used as a calibration sample. Owing to the small cross section of C for He ion, the error on the absolute H content calculated by this method is about 25%. As stated earlier, it should be noticed that such an error on the absolute concentration does not affect any interpretation of the tendency of the H changes. A VEECO Dimension 3100 in tapping mode was employed for the AFM analysis. IR absorbance gave information on how H bonds to Si and Ge before and after annealing. An Oriel Cornerstone instrument was used. Makyoh topography [13] was employed to measure the mean curvature radius of the films to evaluate their stress status using the Stoney formula [13-15]. A Young's modulus and Poisson ratio of 130 GPa [16,17] and 0.28 [16,17], respectively, were assumed for the (100) Si wafer.

## Results and discussion

**Figure 2:**
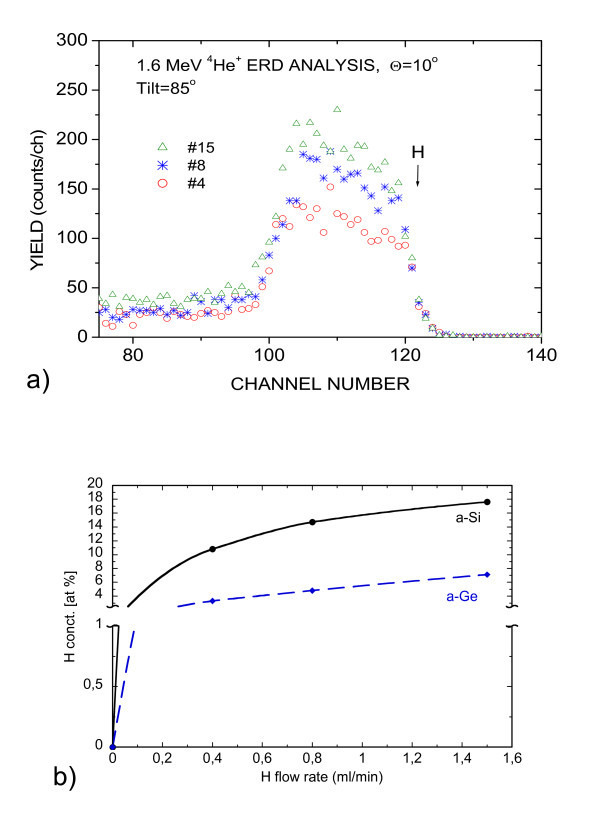
**Calibration of H incorporation with ERDA. (a)** 1.6 MeV ^4^He^+ ^ERDA spectra of H in the a-Si single layers hydrogenated at flow rates of 0.4, 0.8 and 1.5 ml/min (#4, #8 and #15, respectively, in the plot). **(b) **Total H concentration in a-Si (solid black line) and a-Ge (dash blue line) layers as a function of the H flow rate as determined by ERDA.

The calibration of the sputtering apparatus as regards the incorporation of H was done using ERDA by means of the single layers of a-Si and a-Ge. Figure 2a shows the ERDA spectra for the non-annealed a-Si layers hydrogenated at different flow rates. The signal of the recoiled H atoms from the sample surface locates at channel 120. Behind the surface, the distribution of H seems to be reasonably homogeneous in the whole layer. Small peaks at channels 97 and 120 can be associated with the contamination at the surface either of the deposited layer or of the substrate. The tail behind the H peak is due to the multiple scattering, which the RBX code is not yet able to simulate. Similar spectra were obtained for a-Ge. By using the simulation program of ref. [11], the calibration curves of Figure 2b giving the incorporated at.% of H as a function of the H flow rate were obtained. The increase in H concentration in a-Si already tends to slow down significantly between 1 and 1.5 ml/min flow rate (0.78 and 1.46% partial of total pressure), reaching a maximum value of 17 at.%. In a-Ge, the same slowing down trend is observed for the same flow rate values reaching a maximum value of only about 7 at.% (Figure 2b).

**Figure 3:**
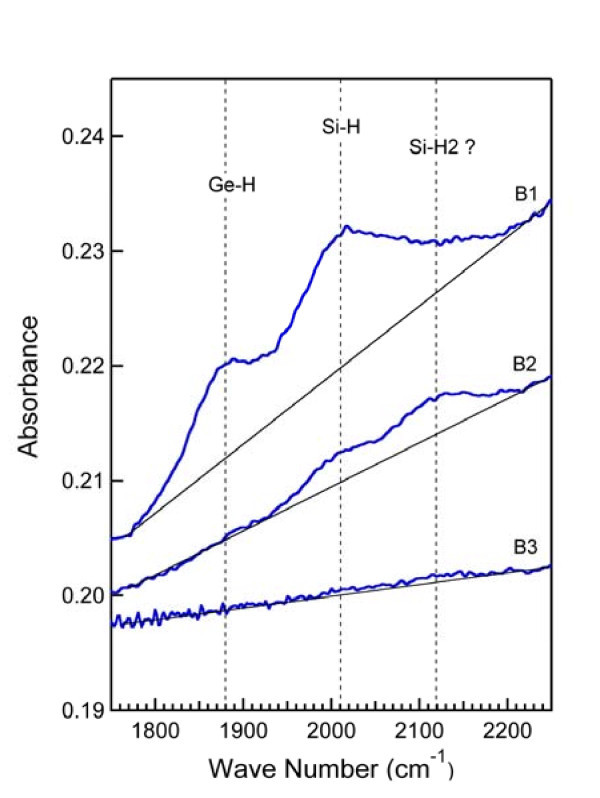
**IR absorbance spectra in the stretching mode range of the wave number for a-Si/a-Ge MLs sputtered under H flow rate of 0.8 ml/min**. B1 is the spectrum of the as-deposited layer, B2 the spectrum after annealing at 400°C for 1 h and B3 the one after annealing at 400°C for 10 h.

A typical set of IR absorbance spectra in the stretching mode range of the wave number is shown in Figure 3. The spectra refer to MLs hydrogenated with a H flow rate of 0.8 ml/min: B1 is the spectrum of the as-deposited layer, B2 the spectrum of the ML annealed at 400°C for 1 h and B3 the one of the sample annealed at 400°C for 10 h. Spectrum B1 shows the peaks at 1880 and at 2010 cm^-1^, which are the fingerprints of the monohydride bonds of H to Ge and Si, respectively [10,18-20]. The shape of the Si-H peak indicates that the peak of the Si di-hydride bond, Si-H_2_, at about 2140 cm^-1 ^could also exist hidden in the tail of the Si-H peak at high wave numbers. The shift with respect to the standard value of 2100 cm^-1 ^can be due to the presence of (Si-H_2_)_*n *_poly-hydrides [10,18] or to a possible contamination of the hydrides by oxygen [18]. The latter contamination, if any, may come from oxygen residues in the sputtering chamber. The presence of the Ge di-hydride on the high wave number side of the Ge-H peak is not certain. The possible existence of Si-H_2 _bonds could be suggested by spectrum B2 also showing a peak around 2140 cm^-1^. Figure 3 shows that, upon annealing, the Si-H and Ge-H bonds break with consequent release of H. H has totally been released from Ge already after 1 h annealing, while it still remains somewhat bound to Si as mono- and di-hydride. After 10 h annealing, H is totally released from Si as well.

**Figure 4:**
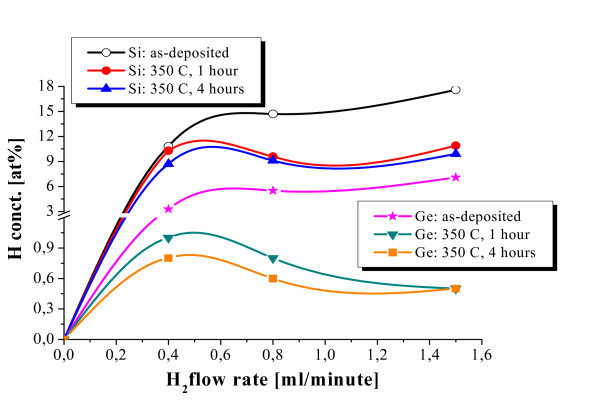
**H concentration, as determined with ERDA, as a function of the H flow rate in a-Si and a-Ge single layers before and after annealing at 350°C for 1 and 4 h**.

**Figure 5:**
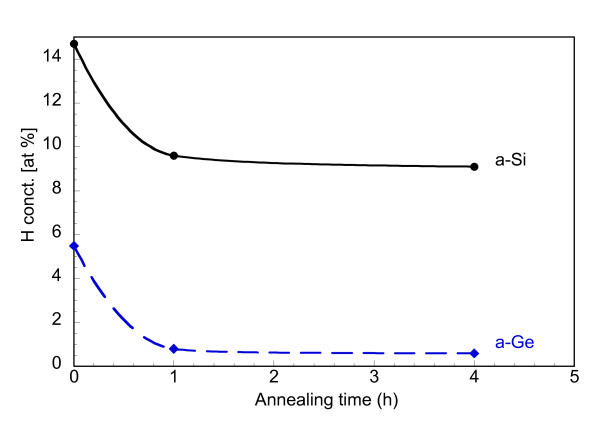
**H concentration, extracted from Figure 4, as a function of the annealing time at 350°C in a-Si (solid black line) and a-Ge (dash blue line) single layers hydrogenated at 0.8 ml/min**.

**Figure 6:**
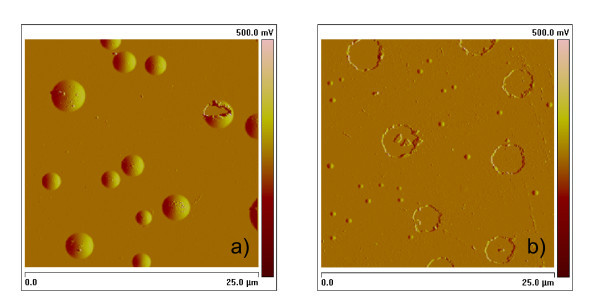
**AFM amplitude image of (a) a-Si and (b) a-Ge single layer after annealing at 350°C for 4 h**. H flow rate 1.5 ml/min. In **(a) **bumps (close bubbles with H still inside) are predominant (only one is broken). In **(b) **large craters, i.e. exploded bumps with escape of H, are by far predominant. Very tiny bumps are also present.

The different release efficiencies of H in a-Si and a-Ge were also studied with ERDA by using the 40-nm-thick single films. The results are summarized in Figure 4 for the case of annealing at 350°C for times of 1 and 4 h. Irrespective of the initial H content (i.e. H flow rate) in the as-deposited films, a decrease in the H concentration upon annealing is observed, which is greater for longer annealing time. However, such a decrease is more effective in the case of the a-Ge film, as can be seen in Figure 5 that compares the decreases in H concentration in the two types of material (sputtered with H flow rate of 0.8 ml/min) as a function of the annealing time at 350°C. In a-Ge, the decrease is 85% with respect to the non-annealed reference sample (from 5.5 to 0.8 at.%) just after 1 h, whereas it is only 35% for a-Si (from 14.7 to 9.6 at.%). By annealing for 4 h only a small further decrease in the H concentration of 3-4% is observed in both Si and Ge. This indicates that the release of H in the a-Ge layer was highly effective, and that its escape from the layer was very fast. Evidence for this is given in Figure 6 which shows the surface morphology of the two types of layer after annealing. For the same annealing time, either 1 or 4 h, the Si layer mainly exhibits surface bumps after annealing, indicating that H is still in the film, though partially gathered in bubbles, whilst the Ge layer exhibits mostly craters, i.e. exploded H bubbles, as deep as the layer, suggesting that nearly all H has escaped in agreement with the ERDA results (Figures 4 and 5). The Si film also contains some broken bumps (one is visible in Figure 6a) which would explain the 35-38% decrease of the H concentration detected by ERDA.

The structure degradation that produces craters is caused by two mechanisms in succession. First, the release of H and the formation of the H bubbles. Second, the creation of craters if the initial H content is very high and/or the annealing conditions are very severe [7-9]. As to the release of H, the above mentioned results are evidence that it is more efficient and faster in the a-Ge layers. This is in agreement with the previous literature according to which the binding energy of the Ge-H bond is smaller than that of the Si-H bond [4,21-24]. In particular, Tsu et al. [24] found that it is 69 kcal/mole for Ge-H and 76 kal/mole for Si-H. The faster release of H in a-Ge would cause a faster increase in the size of the H bubbles to the critical value for their explosion and formation of craters. The results of this study would confirm that the origin of the structural degradation of the MLs of a-Si/a-Ge observed in previous studies (Figure 1 and refs. [7-9]) very likely primarily starts in the Ge layers mostly because of the lower binding energy of H-Ge with respect to H-Si bonds.

It should be noticed that crater formation could also be favoured by intrinsic stresses. The sample stress as measured by MT was always compressive, as found by others [25-27], with values of about 1, 0.15 and 0.33 GPa for as-deposited, non-hydrogenated single layers of a-Si, a-Ge and a-Si/a-Ge MLs, respectively. For a-Si, this result is in reasonable agreement with the literature data [27,28]. Not much is known for a-Ge. As expected, the stress for the MLs is in between those of the single layers. Nickel and Jackson [29] have speculated that the strain released as a consequence of the break of the H-host atom bonds can be re-created by its propagation through the amorphous network to the neighbouring atoms and reconstruction of strained Si-Si bonds. They concluded that the average network strain remains independent of the H concentration and annealing as well [29]. It might thus be assumed that the annealed hydrogenated samples do not change their stress significantly with respect to that measured in the as-deposited ones. Other findings suggest that annealing causes stress relieve in hydrogenated amorphous Si/Ge MLs [30]. Should there be changes in these samples, it is very likely that the intrinsic stress of a-Ge always remains smaller than the one of a-Si upon annealing owing to the great difference between the values of the as-deposited samples. The contribution of stress should thus play a minor role in differentiating the formation rate of the craters in a-Si and a-Ge. Further investigations are underway to better clarify this point.

## Abbreviations

AFM: atomic force microscopy; ERDA: elastic recoil detection analysis; IR: infrared; MLs: multilayers; MT: Makyoh topography.

## Competing interests

The authors declare that they have no competing interests.

## Authors' contributions

CF coordinated the interpretation of the results and wrote the manuscript, MS grew the samples by sputtering and suggested the experiment, NQK performed the ERDA measurements, ACs carried out the sample heating experiments, FR did the Makyoh topography measurements, ZE participated in the coordination-realisation of the IR measurements, LN made the AFM work, DLB participated in the design of the study, H-GB performed the IR measurements.
